# Editorial: Adolescent addictions and risky behaviors: implications for health

**DOI:** 10.3389/fpsyt.2025.1570293

**Published:** 2025-03-20

**Authors:** María Cristina Martínez-Fernández, Paulo Seabra, Elena Fernández-Martínez

**Affiliations:** ^1^ SALBIS Research Group, University of León, Faculty of Health Sciences, León, Spain; ^2^ Nursing Research, Innovation and Development Centre of Lisbon (CIDNUR), Lisbon Nursing School (ESEL), Lisbon, Portugal

**Keywords:** adolescent, cannabis, alcohol, tobacco, internet addiction (IA), addiction

Adolescence is a transitional stage marked by significant cognitive and emotional development, accompanied by crucial psychosocial transitions. During this period the initiation of substance use and engagement in risky behaviours reach its peak (1).

In relation to the use of alcohol, tobacco and cannabis, Villanueva-Blasco et al. have explored the relationship between these substances and various factors associated with the school environment, establishing indirect connections with behavioural problems. The results reveal that behavioural problems are positively correlated with alcohol and the problems associated with its ingestion, while cannabis consumption showed an inverse correlation with these problems. Likewise, a positive association was found between tobacco and cannabis use. Structural equation modelling demonstrated that the perception of the school environment exerts an influence on adolescent behaviour, with the sense of challenge experienced by young people in their school environment directly correlating with the presence of behavioural problems. Additionally, problem behaviour was predicted using alcohol, tobacco and cannabis, as well as excessive alcohol consumption.

In line with this Research Topic, a systematic review examining longitudinal studies on the effect of social networks on adolescent cannabis use has been published by (Torrejón-Guirado et al.). The results suggest that social networks are essential for understanding the impact of cannabis consumption and the mechanisms of peer influence, demonstrating that cannabis use among friends is associated with a higher frequency and intensity of use. Furthermore, the review indicates an increase in consumption when adolescents do not feel close to their schoolmates, friends, or local community environment.

Consequently, studies underscore the significance of environmental influences on the onset of substance use. A study conducted among Chinese adolescents who consume alcohol (Liu et al.) revealed that those with peers who drink are 11.1% more likely to consume alcohol than those without such peers, with this probability being higher among males. A positive correlation has been identified between the presence of siblings and an increased likelihood of alcohol consumption. The absence of parental care has been shown to amplify the impact of peer influence on alcohol use. It is imperative for educational institutions to prioritize the dissemination of preventive education in contexts where young individuals may be more susceptible to peer influences, with the aim of preventing the initiation of substance use (Liu et al., Dadras).

Regarding tobacco use, there is a notable gap in the literature regarding the factors associated with the stages of behaviour change in the Transtheoretical Model that are applicable to smoking cessation among adolescents (Dadras). Consequently, Dadras address this in their study of Indonesian youth, emphasizing the significance of promoting cessation messages, knowledge, and individual attitudes toward quitting at each stage. This is particularly salient when formulating interventions that tackle age-specific barriers, gender disparities, cultural influences, environmental factors, and prevailing attitudes toward smoking. The influence of parents and teachers is critical, having parents who smoke and observing teachers smoking increases the likelihood that adolescents will enter the contemplation and action phases of quitting, respectively. Exposure to cigarette advertisements on television, social media, and at social events, as well as receiving free or discounted cigarettes, has been associated with an increased likelihood of being in both the contemplation and action stages of smoking cessation. Early initiation of cigarette smoking has been associated with an increased likelihood of individuals entering the contemplation and action phases of quitting. Moreover, beginning regular smoking at an early age is linked to a higher risk of developing nicotine dependence and a lower probability of attempting to quit. A systematic review with meta-analysis has demonstrated a cross-sectional relationship between chronic smoking and neurocognitive impairments in adolescents and young adults, notably revealing an association between chronic tobacco use and impaired impulsivity in this population (Elatfy et al.).

Risky behaviours and non-substance addictions must be considered, as problematic internet use has been shown to significantly impact an individual’s physical, mental, and social well-being (2). The study by Demirdöğen et al. highlights that problematic internet use is preceded by negative coping strategies, high levels of escapism, functional impairment, and excessive network usage exceeding seven hours per day. Additionally, escapism levels were found to be higher among youths exhibiting problematic internet use. In this context, research suggests that for adolescents with borderline personality disorder, transdiagnostic online integrative treatment may serve as an effective approach to reducing symptoms of risky behaviours and internet addiction (Mohamadpour and Mohammadi).

In this context, Internet Gaming Disorder (IGD) has been defined as a behavioural addiction characterized by excessive and compulsive video game use (3). Research indicates that adolescents diagnosed with IGD exhibit alterations in resting-state functional connectivity, which are linked to heightened impulsivity and increased disorder severity (Zhao et al.). A qualitative approach is essential when studying this demographic, as gaming addiction may be influenced by cultural factors, early exposure, social networks, and peer pressure (Mazaherizadeh et al.). The concept of “life crafting” has emerged to describe how gaming shapes young individuals’ identities and career aspirations (Mazaherizadeh et al.). Notably, clinically significant age-related disparities have been identified among individuals seeking treatment for gaming disorder. Younger treatment seekers tend to progress more rapidly toward problematic gambling compared to adults (Hofstedt and Gordh). In this study, both age groups exhibited similar levels of psychiatric symptoms, including Attention Deficit Hyperactivity Disorder, Autism Spectrum Disorders, and problem gambling. However, among younger individuals, problematic gambling develops approximately seven years after their initial exposure to gambling, whereas in adults, this progression took around eleven years. These findings suggest a correlation between younger age at treatment-seeking and a faster escalation to gaming disorder.

The manuscripts included in this Research Topic represent a significant step forward in the comprehensive understanding of substance use and addictive behaviours among adolescents. The findings contribute to the essential progress needed to assess the current context and develop interventions aimed at preventing initiation or promoting cessation of use. These results provide valuable insights into the contemporary landscape, facilitating the design of effective intervention strategies. Emphasis is placed on the importance of identifying evidence-based approaches to addressing risk situations. To achieve this, the collaboration of multidisciplinary teams is crucial, ensuring a holistic and integrated approach that encompasses both prevention and treatment.
